# Role of *Saccharomyces cerevisiae* Fcy Proteins and Their Homologs in the Catabolism of Modified Heterocyclic Pyrimidine Bases

**DOI:** 10.3390/microorganisms13071506

**Published:** 2025-06-27

**Authors:** Jaunius Urbonavičius, Iglė Vepštaitė-Monstavičė, Juliana Lukša-Žebelovič, Elena Servienė, Daiva Tauraitė

**Affiliations:** 1 Department of Chemistry and Bioengineering, Faculty of Fundamental Sciences, Vilnius Gediminas Technical University (VILNIUS TECH), Saulėtekio al. 11, 10223 Vilnius, Lithuania; jaunius.urbonavicius@vilniustech.lt (J.U.); juliana.luksa@gamtc.lt (J.L.-Ž.); elena.serviene@gamtc.lt (E.S.); 2 Laboratory of Genetics, State Scientific Research Institute Nature Research Centre, Akademijos Str. 2, 08412 Vilnius, Lithuania; igle.vepstaite-monstavice@gamtc.lt

**Keywords:** *Saccharomyces cerevisiae*, modified heterocyclic base, modified uracil, modified cytosine, Fcy proteins, transport, permease, catabolism

## Abstract

The synthesis of various heterocyclic base modifications of nucleic acids has been thoroughly investigated; however, much less is known about their catabolism. Also, little is known about the transport of such compounds across the microbial cell membranes. Using the *Saccharomyces cerevisiae* single-gene deletion library, we performed genome-wide screening for genes affecting the growth of yeast in minimal media supplemented with *N*^4^-acetylcytosine as a source of uracil. We found that Fcy1, Fcy21, Bud16, Gnd1, and Fur4 proteins are required for efficient growth in the tested medium. Additionally, we used several heterocyclic pyrimidine bases and Fcy homolog mutants to test their growth in respective minimal media. We found that tested permeases differently affect the growth of yeast that is dependent on the heterocyclic pyrimidine bases used as a source of uracil. The most pronounced effect was observed for the ∆*fur4* mutant, which was growing much slower than the corresponding wild-type strain in the media supplemented with *N*^4^-acetylcytosine, 4-methylthiouracil, *N*^4^-methylcytosine, *N*^4^,*N*^4^-dimethylcytosine, 2-thiouracil, or 4-thiouracil. We suggest that Fur4 protein is the major yeast transporter of modified heterocyclic pyrimidine bases. Our observations might be helpful when investigating the actions of various heterocyclic base-based antifungal, anticancer, and antiviral drugs.

## 1. Introduction

Among the different species of nucleic acids that are present in cells, transfer RNA contains the highest number of chemical modifications, with more than 170 different species found [1,2,3]. These modifications are important for the maintenance of tRNA’s structure, stability, localization, cellular transport, and translation. The role of modified nucleosides in different nucleic acid species has been extensively investigated, including their roles in diseases and the production of mRNA vaccines [4,5,6,7,8]. The introduction of the chemical groups and the respective enzymes catalyzing such reactions have been widely investigated. Still, much less is known about the degradation and return to metabolism of modified nucleosides and the corresponding heterocyclic bases. The demodification of the nucleic acids is often studied at the DNA or RNA level as part of epigenetics studies [9,10,11]. However, such enzymes acting on the small molecules produced by the action of the nucleases are much less investigated despite their possible roles in drug biosynthesis and metabolism. Indeed, different pyrimidine and purine analogs act as medicines in the treatment of various diseases. Probably the best known of such drugs is 5-fluorocytosine, known under different brand names (like Flucytosine and Antocil) and first synthesized almost 70 years ago [12,13]. Also, different analogs of the nucleosides, such as Entecavir, Floxuridine, Capecitabine, Nelarabine, Remdesivir, and many others, are known for their antifungal, antiviral, and anticancer properties. These compounds are FDA-approved or in clinical development [14,15,16]. Therefore, studying the enzymes that produce modified heterocyclic bases is important due to their possible roles in the biosynthesis of such antiviral and anticancer drugs [17,18].

Budding yeast *Saccharomyces cerevisiae* is a eukaryotic model microorganism used for studies of various cellular functions. In this work, we have explored the role of pyrimidine permeases in supporting the growth of the modified uracil and cytosine nucleobases. It is known that the transportation of uracil, but not cytosine or thymine (5-methyluracil), is mediated by Fur4 uracil permease [19,20]. Instead, cytosine uptake is mediated by purine–cytosine permease Fcy2 protein [21]. The *fcy2* null mutants are viable but not able to take up cytosine or 5-methylcytosine [22]. In addition, this mutant becomes, to varying degrees, resistant to anticancer drugs 5-fluorocytosine, 5-fluorouracil, cisplatin, and doxorubicin [23,24]. It was demonstrated that *fcy2* mutants exhibit low-level, dose-dependent toxicity to 5-fluorocytosine, whereas *fcy1* mutants (deficient in the cytosine deaminase that is essential for conversion of 5-fluorocytosine to 5-fluorouracil) display strong dose-independent resistance [25]. In that work, a search for additional genes that could mediate the toxicity of 5-fluorocytosine led to the discovery of several cytosine permease homologues (*FCY21*, *FCY2*, *FUR4*, *DAL4*, and *YOR071C* (*NRT1*)). Recently, several paralogs of cytosine permease (*FCY2*, *FCY3*, and *FCY4*) and cytosine deaminase (*FCY1*, *FCY5*, and *FCY6*) were detected in *Cryptococcus* yeast, the etiologic agent of cryptococcosis [26]. There, it was demonstrated that the function of cytosine permeases can vary depending on the nitrogen source. For example, Fcy3 acts as a secondary permease in *Cryptococcus gattii* when the amino acids arginine, asparagine, or proline are used. Despite the structural similarity of the modified uracil and cytosine bases to the unmodified ones or their fluorinated counterparts, the involvement of Fcy proteins and their homologs in transportation across the yeast membrane has not been systematically investigated. In this work, we used the uracil auxotrophy-based *S. cerevisiae* screening system to investigate the role of such transporters.

## 2. Materials and Methods

### 2.1. Synthesis of Modified Pyrimidine Heterocyclic Bases

General Information

Chemicals, reagents, and solvents were purchased from Sigma-Aldrich (Merck group, Darmstadt, Germany) and Thermo Fisher Scientific (Vilnius, Lithuania) and used without further purification. 4-Methylthiouracil, *N*^4^-methylcytosine, and *N*^4^,*N*^4^-dimethylcytosine were synthesized as described previously [27]. Other modified heterocyclic bases were synthesized by adapting and modifying methods reported in the literature, including 2-methylthiouracil [28], 2-methylthiocytosine [29], and 3-methyluracil [30]. The synthesis of these modified compounds is described below. Thin layer chromatography (TLC) was carried out on TLC Silica gel 60 F_254_ (Merck, Darmstadt, Germany) and column chromatography on silica gel 60 (0.063–0.200 mm) (Merck, Darmstadt, Germany) using chloroform/methanol solutions as a mobile phase. NMR spectra were recorded in deuterated dimethylsulfoxide on an Ascend 400 ^1^H NMR—400 MHz and ^13^C NMR—101 MHz (Bruker BioSpin, Rheinstetten, Germany). Chemical shifts (Δ) were reported in ppm relative to the solvent resonance signal as an internal standard. The high-performance liquid chromatography (HPLC) was performed using the UFLC LC-20AD system equipped with a photo diode array detector (Shimadzu, Kyoto, Japan). The chromatographic separation was conducted using a Symmetry C18 column, 4.6 × 75 mm (Waters, Milford, MA, USA), at 40 °C and a mobile phase that consisted of 5 mM ammonium acetate buffer (solvent A) and methanol (solvent B).

#### 2.1.1. Synthesis of 2-Methylthiouracil

To a solution of 2-thiouracil (150 mg, 1.17 mmol) and 1 M NaOH (1.17 mL) in 50% ethanol (6.0 mL), methyl iodide (144 μL, 2.34 mmol) was added. The mixture was stirred at room temperature for 2 h. After the reaction was completed (TLC), the mixture was neutralized with diluted acetic acid (25% in water) to pH 6. After evaporation under reduced pressure, the residue was purified through flash column chromatography (silica gel, chloroform/methanol mixture, 10/0→10/1). Yield 120 mg (72%), yellowish foam, Rf = 0.56 (CHCl_3_/MeOH–9/1). HPLC purity 98%. ^1^H NMR (DMSO-d6, 400 MHz): Δ = 2.48 (s, 3H, CH_3_), 6.10 (s, 1H, CH=CH), 7.86 (s, 1H, CH=CH), 12.73 (s, 1H, NH). ^13^C NMR (DMSO-d6, 101 MHz): Δ = 13.34, 105.73, 142.53, 161.43, 176.56.

#### 2.1.2. Synthesis of 2-Methylthiocytosine

To a solution of 2-thiocytosine (160 mg, 1.26 mmol) and NH_4_OH (2 mL, 25%) in 50% methanol/water (6.0 mL), CH_3_I (236 µL, 3.78 mmol) was added, and the mixture was stirred at room temperature for 1.5 h. After the reaction was completed (TLC), the solvents were evaporated under reduced pressure, and the residue was purified through flash column chromatography (silica gel, chloroform/methanol mixture, 10/0→10/0.5). Yield 160 mg (90%), white solid, Rf = 0.6 (CHCl_3_/MeOH–9/1). HPLC purity 97%. ^1^H NMR (DMSO-d6, 400 MHz): Δ = 2.38 (s, 3H, CH_3_), 6.13 (d, *J* = 5.8 Hz, 1H, CH=CH), 6.91 (s, 2H, NH_2_), 7.90 (d, *J* = 5.8 Hz, 1H, CH=CH). ^13^C NMR (DMSO-d6, 101 MHz): Δ = 13.61, 101.56, 155.28, 163.46, 170.56.

#### 2.1.3. Synthesis of 3-Methyluracil

(i) Protection of N-1 with BOC-group: To a solution of uracil (0.224 g, 2 mmol) and (*t*-BOC)_2_O (0.436 g, 2 mmol) in acetonitrile (5 mL), DMAP (2.2 mg, 0.02 mmol) was added, and the mixture was stirred at room temperature for 3 h. The solvent was removed under vacuum, and the residue was used in the next step without purification.
(ii) N-3 methylation: The crude material from the previous (i) step was diluted with DMF (10 mL), and NaH (120 mg, 3.1 mmol, 60% in mineral oil) was added. The suspension was stirred at room temperature for 30 min, and then CH_3_I (194 µL, 3.1 mmol) was added. The reaction mixture was stirred for 2 h and then poured into cold water (20 mL), and the product was extracted with ethyl acetate (3 × 20 mL). The extracts were dried (Na_2_SO_4_), evaporated to dryness, and used in the next step.
(iii) N-1 BOC deprotection: The crude material was dissolved in chloroform (10 mL), and Trifluoroacetic acid (1.5 mL, 19.6 mmol) was added. The reaction mixture was stirred at room temperature for 24 h. After the reaction was completed (TLC), the solvent was evaporated under reduced pressure, and the residue was purified through flash column chromatography (silica gel, chloroform/methanol mixture, 10/0→10/0.5). Overall yield 100 mg (40%), white solid, Rf = 0.48 (CHCl_3_/MeOH–9/1). HPLC purity 97%. ^1^H NMR (DMSO-d6, 400 MHz): Δ = 3.11 (s, 3H, CH_3_), 5.59 (dd, *J* = 7.6, 1.1 Hz, 1H, CH=CH), 7.44 (dd, *J* = 7.5, 5.9 Hz, 1H, CH=CH), 11.13 (s, 1H, NH). ^13^C NMR (DMSO-d6, 101 MHz): Δ = 26.86, 100.02, 140.84, 152.07, 163.77.

### 2.2. Yeast Strains and Growth Media

Experiments were performed with a YKO (Yeast Knockout) collection of *Saccharomyces cerevisiae* strains (BY4741 background, *MAT*a, *his3*Δ*1*, *leu2*Δ*0*, *met15*Δ*0*, *ura3*Δ*0*), where single ORFs were replaced by the KanMX4 module (4784 strains in total, Thermo Scientific Molecular Biology, Lafayette, CO, USA).

Yeast strains were grown in the standard yeast peptone dextrose agar medium (YPD): 1% yeast extract, 2% peptone, 2% dextrose, and 2% agar. For screening purposes, minimal dextrose agar medium (MD) containing 2% glucose, 1% (NH_4_)_2_SO_4_, 0.09% KH_2_PO_4_, 0.05% MgSO_4_, 0.023% K_2_HPO_4_, 0.01% CaCl_2_, 0.01% NaCl, and 2.5% agar, supplemented with 0.002 µg/mL of biotin, 0.5 µg/mL of *β*-alanine, and 0.2 µg/mL of thiamine, was used [31]. Uracil, cytosine, or modified pyrimidines (4-methylthiouracil, 2-methylthiouracil, 2-methylthiocytosine, *N*^4^-methylcytosine, 3-methyluracil, *N*^4^,*N*^4^-dimethylcytosine, 2-thiouracil, 4-thiouracil, and *N*^4^-acetylcytosine) at a final concentration 20 µg/mL were added to the MD synthetic medium.

### 2.3. Genome-Wide Screening Procedure

*S. cerevisiae* YKO library strains (BY4741 and strains containing single deletions) were arrayed in 96 colony formats on YPD agar plates and transferred onto the MD–agar plates supplemented with uracil or *N*^4^-acetylcytosine. After incubation for 3–4 days at 30 °C, the size of the colonies grown on the media with the different additives was assessed and compared to each other. MD plates that contained uracil were used as controls. Yeast strains growing differently on MD–agar plates containing uracil and *N*^4^-acetylcytosine were selected for further studies.

### 2.4. Growth of Selected YKO Mutants and Retesting on MD–Agar Medium

For growth analysis, yeast strains were streaked on MD–agar media containing uracil and *N*^4^-acetylcytosine and grown for 3–4 days at 30 °C. For verification of yeast detected in screening, single-deletion yeast strains were grown overnight at 30 °C in liquid YPD medium. Yeast cells were collected through centrifugation for 2 min at 10,000× *g* and washed twice with 0.9% NaCl to reach a final concentration of OD_600_ = 1. The serial dilutions were performed (from 10^1^ to 10^7^ fold), and 5 µL of yeast suspension was drop-seeded onto MD–agar medium with additives. Yeast growth on the different media was compared after incubation for 3–4 days at 30 °C.

### 2.5. Determination of the Growth Rate of Selected YKO Mutants

Yeast strains were grown in YPD medium at 30 °C overnight. Yeast cells were washed twice with liquid MD medium and diluted to 0.2 OD_600_ with MD with additives. Yeast suspensions (200 µL) were poured into 96-well microplates, and the OD_600_ was measured at 30 °C with shaking using a TECAN Infinite M plex absorbance microplate reader. Data were parsed and grouped into samples based on replicate wells. Data were processed in Python 3.11 (version 3.11, Python Software Foundation, Wilmington, DE, USA, 2024) using NumPy 1.26 [32] and SciPy 1.13 [33]. Automatic detection of the exponential (log) growth phase was performed using a moving window of 7 consecutive timepoints (~140 min). The growth rate constant (μ) was determined as the slope of the natural logarithm of OD_600_ versus time. The generation time (G) was calculated as ln(2)/μ. Results were aggregated across replicates for each sample, and the mean and standard error of the mean (SEM) were calculated. For statistical comparisons between the two medium conditions (uracil vs. *N*^4^-acetylcytosine) within each strain, one-way ANOVA was performed on the replicate growth rates using the scipy.stats.f_oneway function. Differences were considered statistically significant at *p* < 0.05.

## 3. Results and Discussion

**Figure 1:**
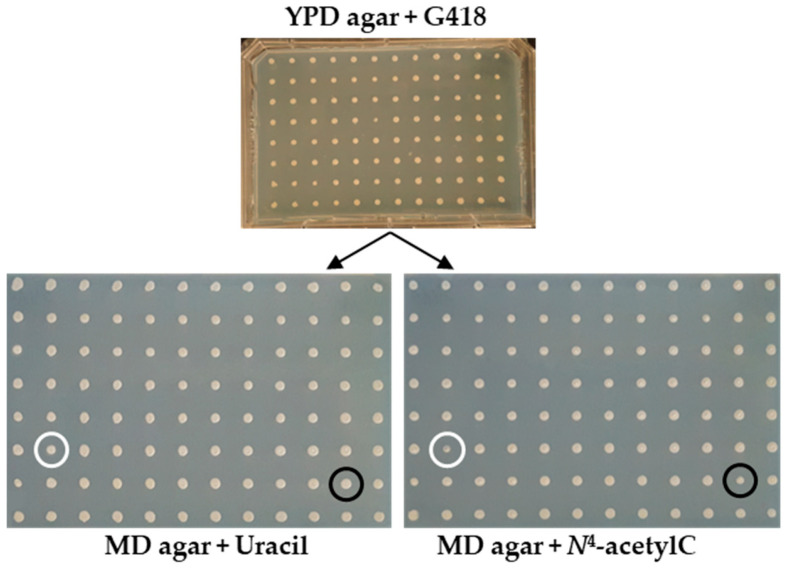
Genome-wide screening procedure using the Yeast Knockout (YKO) collection on MD–agar plates with uracil and, similarly, with *N*^4^-acetylcytosine instead of uracil. Potential candidates involved in the demodification of cytosine derivative (*N*^4^-acetylcytosine) were selected based on the different growth of colonies on MD–agar supplemented with uracil or with *N*^4^-acetylcytosine. Circles of the same color (white or black) indicate the growth phenotype of individual yeast deletion mutants on each respective medium.

**Figure 2:**
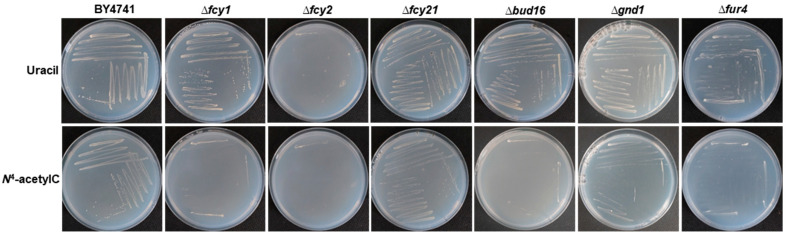
Growth of *S. cerevisiae* mutants identified in the primary screen in MD medium supplemented with either uracil or *N*^4^*-*acetylcytosine.

**Figure 3:**
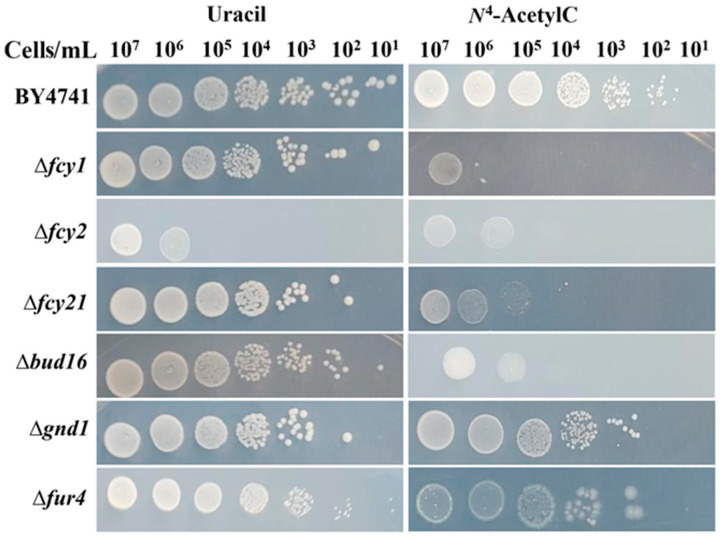
Growth of *S. cerevisiae* uracil auxotroph in MD medium supplemented with either uracil or *N*^4^*-*acetylcytosine after serial dilutions.

As the first attempt to identify the gene(s) involved in the demodification of cytosine derivatives, thereby supporting the growth of uracil auxotrophs in minimal media, we selected *N*^4^-acetylcytosine and performed genome-wide screening using this compound as the substrate. It is known that *N*^4^-acetylcytosine supports the growth of *Escherichia coli* uracil auxotrophs via conversion of it into cytosine and further into uracil. It was found that the hydrolysis of *N*^4^-acetylcytosine is catalyzed by the YqfB protein [34]. However, protein–protein BLAST+ 2.16.0 analysis of the YqfB sequence against the *S. cerevisiae* S288C translated genome demonstrated no significant similarities. Still, similarly to *E. coli*, *N*^4^-acetylcytosine significantly supported the growth of the *S. cerevisiae* Δ*ura3* (Δ*pyrF*) strain (Figure 1, Figure 2 and Figure 3). Therefore, we took a similar approach to the previously used genome-wide screening strategy to identify the genes of *S. cerevisiae* potentially involved in the conversion of *N*^4^-acetylcytosine into uracil. We expected that the growth rate of certain mutants would be affected under selective conditions, allowing us to detect relevant candidates through comparative growth.

The screening system utilized a library of *S. cerevisiae* non-essential gene deletion mutants in the BY4741 (Δ*ura3*) background. These mutants were spotted onto MD medium supplemented with the methionine, leucine, histidine, and vitamin solution and *N*^4^-acetylcytosine as the sole source of uracil. The primary screen identified 30 candidates exhibiting reduced colony size compared to the controls (Table 1, Figure 1).

After the primary screen, the candidate strains were further evaluated using the drop-seeding method onto MD–agar plates. The verification step confirmed that five mutants—Δfcy1, Δ*fcy21*, Δ*bud16*, Δ*gnd1*, and Δ*fur4*—exhibited impaired growth on MD medium with *N*^4^-acetylcytosine (Figure 2). In addition, the growth of Δ*fcy2* was tested, and it was demonstrated that even when uracil was used, growth was severely affected.

A qualitative growth analysis using serial dilutions was performed for these mutants. Figure 3 demonstrates that growth was variably reduced across the different strains when *N^4^*-acetylcytosine was used as the sole uracil source.

To quantitatively assess growth performance, we measured and compared the growth rates of the five validated mutants in liquid MD medium supplemented with either uracil or *N^4^*-acetylcytosine. Statistical analysis of the growth rate constants (Table 2) revealed that all five mutants exhibited significantly reduced growth in the presence of *N^4^*-acetylcytosine compared to uracil (*p* < 0.05). One of the tested mutants, Δ*gdn1* (ΔYHR183W), displayed a reduced growth rate compared to the wild-type strain. This mutant lacks the enzyme 6-phosphogluconate dehydrogenase, which catalyzes the oxidative reduction of NADP^+^ to NADPH [35]. In this mutant strain, the (NADPH/NADP^+^)/ratio is reduced by about 27% [36]. In the same study, the authors also reported a modest decrease in the pyridoxine (vitamin B_6_) level in the Δ*gdn1* mutant. These results correlate with the finding that deletion of the *BUD16* (YEL029C) gene, which encodes the putative pyridoxal kinase, an enzyme involved in pyridoxal 5-phosphate synthesis, also leads to reduced growth in the medium supplemented with *N*^4^-acetylcytosine. We found that only *FCY1* cytosine deaminase has a clearly defined function, which is likely the deamination of cytosine obtained through the action of yet unknown yeast *N*^4^-acetylcytosine hydrolase. It was demonstrated that deletion of *FCY1* leads to very slow growth in minimal medium supplemented with *N*^4^-acetylcytosine. In addition, detection through the screening of *FCY2*, *FCY21*, and *FUR4* genes, which are all annotated in the *Saccharomyces* Genome Database (SGD, https://www.yeastgenome.org/, accessed on 15 April 2025) as encoding the purine–cytosine–permease, putative purine–cytosine permease, and uracil permease, respectively, indicates that all of these proteins are involved in the transportation of *N*^4^-acetylcytosine.

**Figure 4:**
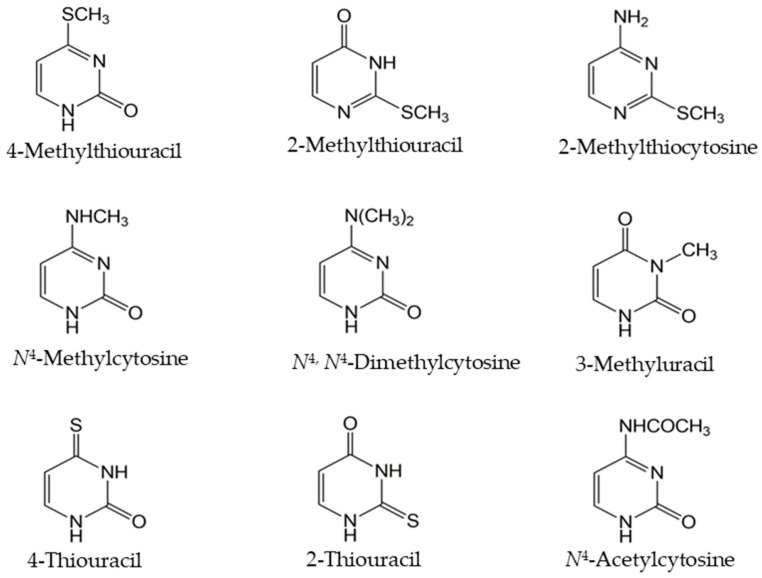
Chemical structure of modified pyrimidines that were used as substrates for the growth of uracil auxotrophs.

Involvement of Fcy1 deaminase and Fcy2/Fcy21/Fur4 permeases in the catabolism of the *N*^4^-acetylcytosine prompted us to investigate the role of other *Fcy* proteins and their homologs in this process. For this purpose, we tested several additional mutants of cytosine/adenine permease homologs that were previously demonstrated to be involved in the toxicity of 5-fluorocytosine [25]. Namely, in addition to those described in the screen above, Δ*fcy1*, Δ*fcy2*, Δ*fcy21*, and Δ*fur4* mutants and Δ*fcy22*, Δ*dal4*, Δ*fui1*, and Δ*nrt1* strains were tested. As substrates that could potentially support the growth of uracil auxotrophs, 4-methylthiouracil, 2-methylthiouracil, 2-methylthiocytosine, *N*^4^-methylcytosine, 3-methyluracil, *N*^4^,*N*^4^-dimethylcytosine, 2-thiouracil, 4-thiouracil, and *N*^4^-acetylcytosine (Figure 4) were tested. The growth phenotypes of all tested mutants on these substrates are summarized in Table 3.

Our findings indicate that to support the growth of yeast uracil auxotrophs, *N*^4^-acetylcytosine is first converted into cytosine and then subsequently into uracil. This is consistent with data obtained for *E. coli*, where the enzymatic activity of YqfB amidohydrolase towards *N*^4^-acetylcytosine was demonstrated [34]. Surprisingly, ∆*fcy2* or ∆*fcy22***,** but not ∆*fcy21* mutants, severely affected yeast growth not only for *N*^4^-acetylcytosine but also several other pyrimidines tested, including uracil and cytosine. However, it is important to note that 2-methylthiouracil, 2-methylthiocytosine, and 3-methyluracil support equally pure growth of the corresponding wild-type BY4741 strain. Given the similarly weak growth observed for these and other mutants, the specific roles of individual permeases in the transportation of tested compounds are difficult to judge, and this will not be further discussed. However, in the case of Fcy21, it has a significant role in the transportation of cytosine, *N*^4^-acetylcytosine, and 4-methylthiouracil and a less pronounced role in that of 2-thiouracil and 4-thiouracil. It was previously suggested that Fcy21 is similar to Fcy2, but it cannot substitute for its function [37]. The functionality of Fcy21 and Fcy22 permeases was confirmed by the finding that in the clinical isolates of *Candida albicans*, amino acid substitutions in Fcy21 and Fcy22 were resistant to 5-fluorouracil [38]. Our results suggest that Fcy2, Fcy21, and Fcy22 could have different specificity towards the transportation of various modified pyrimidine bases tested.

One of the investigated Fcy2 homologs, Dal4 protein, is known as allantoine permease [39], which is structurally somewhat similar to the modified pyrimidines. It was found that deletion of the corresponding gene affects yeast growth in the minimal medium supplemented with 4-methylthiouracil and especially *N*^4^,*N*^4^-dimethylcytosine. These findings suggest the role of Dal4 protein in the transportation of these compounds. Another Fcy2 homolog, Fui1 protein, is uridine permease that is not involved in uracil transportation [40]. However, ∆*fui1* mutant grew slower than the corresponding wild-type BY4741 strain in the media supplemented with *N*^4^,*N*^4^-dimethylcytosin but not the other modified pyrimidines tested. This observation suggests that Fui1p might be involved in the transportation of pyrimidines with bulky side chains.

Another transporter that was investigated in this work is uracil permease Fur4 [41,42]. We have demonstrated that the growth rate of the Δ*fur4* mutant was severely affected compared to the corresponding wild-type BY4741 strain in MD minimal media supplemented with either *N*^4^-acetylcytosine, 4-methylthiouracil, *N*^4^-methylcytosine, *N*^4^,*N*^4^-dimethylcytosine, 2-thiouracil, or 4-thiouracil. These findings suggest that Fur4 permease is a major transporter of modified uracil/cytosine bases in yeast. Finally, the nicotinamide riboside transporter Nrt1 [43] has little effect on the translocation of the compounds tested because the corresponding ∆*nrt1* mutation had only a minor effect on growth in minimal media, with a slight decrease of growth in MD supplemented with either *N*^4^,*N*^4^-dimethylcytosine or 2-thiouracil but not the other compounds tested.

Taken together, our results demonstrate that *S. cerevisiae* is capable of utilizing several natural modified heterocyclic pyrimidine bases as a source of uracil to support its growth. This feature might be especially important in minimal media and/or environmental conditions with limited growth resources. Several transporters are involved in the transportation of such heterocyclic bases, yet the Fur4 protein appears to be the major one in this process. Once inside of the cell, modified cytosine and/or uracil bases are converted into the uracil by (yet) unknown enzymes. The discovery of such enzymes may lead to the chemoenzymatic synthesis of novel heterocyclic base analogs with potential antifungal, anticancer, and antiviral activities. Our obtained knowledge also identifies the potential hotspots of mutagenesis associated with the transportation of such drugs across the yeast membrane and resistance to them.

## 4. Conclusions

In this work, we have investigated the role of several Fcy proteins and their homologs in the catabolism of modified heterocyclic pyrimidines in *S. cerevisiae*. The genome-wide screen demonstrated the involvement of Fcy1, Fcy21, Bud16, Gnd1, and Fur4 proteins in supporting the growth of yeast in minimal medium supplemented with *N*^4^-acetylcytosine. Out of these, Bud16 and Gnd1 are involved in the metabolism of pyridoxine (vitamin B_6_), while Fcy1 is cytosine deaminase that is involved in the conversion of *N*^4^-acetylcytosine into uracil. Also, Fcy21 and Fur4 are respective (putative) purine–cytosine and uracil permeases and appear to participate in substrate transport. Testing of the role of those initially found in the genome-wide screen and several other Fcy homologs, namely, Fcy1, Fcy2, Fcy21, Fcy22, Dal4, Fui1, Fur4, and Nrt1 proteins for the growth of yeast in minimal media supplemented either with 4-methylthiouracil, 2-methylthiouracil, 2-methylthiocytosine, *N*^4^-methylcytosine, 3-methyluracil, *N*^4^,*N*^4^-dimethylcytosine, 2-thiouracil, 4-thiouracil, or *N*^4^-acetylcytosine, demonstrated various degrees of involvement, with Fur4 having the most pronounced effect. Taken together, our results suggest that even though different Fcy proteins and their homologs could have an effect on the transportation of modified heterocyclic pyrimidine bases across the membrane, Fur4 is the most important one because, in addition to *N*^4^-acetylcytosine, it apparently affects the translocation of at least 4-methylthiouracil, *N*^4^-methylcytosine, *N*^4^,*N*^4^-dimethylcytosine, 2-thiouracil, and 4-thiouracil. Our observations might be helpful when investigating the actions of various heterocyclic base-based antifungal, anticancer, and antiviral drugs.

## Figures and Tables

**Table 1:** Yeast YKO strains selected from genome-wide screening (primary screen) and following retesting. Growth conditions included MD + U-MD–agar medium with uracil or MD + *N*^4^-AcetylC-MD–agar medium with *N*^4^-acetylcytosine. Growth phenotypes of primary screen: G++ comparable to control yeast colony, G+ slightly reduced yeast colony growth, G− no growth, and G+/− decreased yeast colony growth. The results of retesting are shown according to yeast growth after serial dilutions: +++ 10^7^ dilution, +++/− 10^6^, ++ 10^5^, ++/− 10^4^, + 10^3^, and +/− 10^2^.

		Primary Screen		Retesting		
ORF ID	Gene Name	MD + U	MD + *N*^4^-AcetylC	MD + U	MD + *N*^4^-AcetylC	Description
**YPR062W**	***FCY1***	**G++**	**G+/−**	**+++**	**+/−**	**Cytosine deaminase**
**YER060W**	***FCY21***	**G++**	**G+**	**+++/−**	**++/−**	**Putative purine–cytosine permease**
**YEL029C**	***BUD16***	**G++**	**G+/−**	**+++**	**+/−**	**Putative pyridoxal kinase**
**YHR183W**	***GND1***	**G++**	**G+**	**+++/−**	**++**	**6-phosphogluconate dehydrogenase (decarboxylating)**
**YBR021W**	***FUR4***	**G++**	**G+**	**+++**	**++**	**Plasma membrane localized uracil permease **
YER056C	*FCY2*	G+	G+/−	+/−	+/−	Purine–cytosine permease
YBL067C	*UBP13*	G++	G+	+++/−	+++/−	Ubiquitin-specific protease that cleaves Ub–protein fusions
YBR159W	*IFA38*	G++	G+	+++/−	+++/−	Microsomal beta-keto-reductase
YCR023C		G++	G+	+++/−	+++/−	Vacuolar membrane protein of unknown function
YDL201W	*TRM8*	G++	G+	+++/−	+++/−	Catalytic subunit of a tRNA methyltransferase complex
YDR370C	*DXO1*	G++	G+	+++	+++	Cytoplasmic 5′ exoribonuclease
YDR369C	*XRS2*	G++	G+	+++	+++	FHA domain-containing component of the Mre11 complex
YDR227W	*SIR4*	G++	G+	+++/−	+++/−	SIR protein involved in the assembly of silent chromatin domains
YER005W	*YND1*	G++	G+/−	+++	+++	Apyrase with wide substrate specificity
YDR452W	*PPN1*	G++	G+/−	+++	+++	Dual endo- and exopolyphosphatase with a role in phosphate metabolism
YDR482C	*CWC21*	G++	G+	+++/−	+++/−	Protein involved in RNA splicing by the spliceosome
YIR032C	*DAL3*	G++	G+	+++/−	+++/−	Ureidoglycolate lyase
YJL133W	*MRS3*	G++	G+	+++/−	+++/−	Iron transporter; mediates Fe^2+^ transport across the inner mito membrane
YLR363C	*NMD4*	G++	G+	+++	+++	Protein that may be involved in nonsense-mediated mRNA decay
YLR376C	*PSY3*	G++	G+	+++	+++	Component of the Shu complex (aka PCSS complex)
YLR375W	*STP3*	G++	G+	+++/−	+++/−	Zinc-finger protein of unknown function
YMR068W	*AVO2*	G++	G+	+++/−	+++/−	Subunit of TORC2, a regulator of plasma membrane (PM) homeostasis
YMR105C	*PGM2*	G++	G−	+++/−	+++/−	Phosphoglucomutase
YMR121C	*RPL15B*	G++	G+	+++/−	+++/−	Ribosomal 60S subunit protein L15B
YMR120C	*ADE17*	G++	G+	+++/−	+++/−	Enzyme of ‘de novo’ purine biosynthesis
YNL307C	*MCK1*	G++	G+	+++/−	+++/−	Dual-specificity ser/thr and tyrosine protein kinase
YNL219C	*ALG9*	G++	G+	+++	+++	Mannosyltransferase, involved in N-linked glycosylation
YNR032W	*PPG1*	G++	G+	++	++	Serine protein phosphatase involved in the formation of the FAR complex
YOL009C	*MDM12*	G++	G+	+++/−	+	Mitochondrial outer membrane protein, ERMES complex subunit
YBL042C	*FUI1*	G++	G+	+++/−	+++/−	High-affinity uridine permease; localizes to the plasma membrane

**Table 2:** Growth rate constant and generation time of yeast mutant strains in liquid MD medium supplemented with uracil or *N*^4^-acetylcytosine. The values are means ± SEM from three experiments. Statistical significance between medium conditions (uracil vs. *N*^4^-acetylcytosine) for each strain was determined through one-way ANOVA; * indicates *p* < 0.05.

Sample	Growth Rate Constant, h^−1^	Generation Time, h
BY4741 + uracil	0.103 ± 0.001	6.756 ± 0.080
BY4741 + *N*^4^-acetylC	0.077 ± 0.017	9.794 ± 1.868
Δ*fcy*1 + uracil	0.133 ± 0.006 *	5.237 ± 0.258
Δ*fcy*1 + *N*^4^-acetylC	0.025 ± 0.002	27.965 ± 1.631
Δ*fcy*2 + uracil	0.029 ± 0.002 *	24.275 ± 1.336
Δ*fcy*2 + *N*^4^-acetylC	0.018 ± 0.005	42.172 ± 8.479
Δ*fcy*21 + uracil	0.066 ± 0.001 *	10.594 ± 0.167
Δ*fcy*21 + *N*^4^-acetylC	0.023 ± 0.002	30.103 ± 2.252
Δ*bud16* + uracil	0.052 ± 0.016 *	16.929 ± 6.105
Δ*bud*16 + *N*^4^-acetylC	0.030 ± 0.003	23.688 ± 1.910
Δ*gnd*1 + uracil	0.053 ± 0.014 *	16.441 ± 6.157
Δ*gnd*1 + *N*^4^-acetylC	0.020 ± 0.001	34.540 ± 1.601
Δ*fur*4 + uracil	0.066 ± 0.003 *	10.494 ± 0.533
Δ*fur*4 + *N*^4^-acetylC	0.020 ± 0.002	34.492 ± 3.310

**Table 3:** Growth of yeast *FCY* mutants and their homologs in MD medium supplemented with modified uracil bases. The results are shown according to yeast growth after serial dilutions: +++ 10^7^ dilution, +++/− 10^6^, ++ 10^5^, ++/− 10^4^, + 10^3^, +/− 10^2^.

ORF ID	WT	YPR062W	YER060W	YER056C	YER060W-A	YIR028W	YBL042C	YBR021W	YOR071C
Compound/gene		∆fcy*1*	∆*fcy21*	∆*fcy2*	∆*fcy22*	∆*dal4*	∆*fui1*	∆*fur4*	∆*nrt1*
Uracil	+++	+++	+++/−	+/−	+/−	+++/−	+++/−	+++	+++/−
Cytosine	+++	+++/−	++	+/−	+/−	+++/−	+++/−	+++/−	+++/−
*N*^4^-Acetylcytosine	+++	+/−	++/−	+/−	+/−	+++/−	+++/−	++	+++
4-Methylthiouracil	+++/−	+	+	+/−	+/−	++	+++/−	+/−	+++/−
*N*^4^-Methylcytosine	+++/−	+++/−	+	+/−	+/−	+++/−	+++/−	+/−	+++/−
*N*^4^,*N*^4^-Dimethylcytosine	+++/−	++	++/−	+/−	+/−	+	++/−	+/−	++/−
2-Thiouracil	+++/−	+++/−	++	+/−	+/−	+++/−	+++/−	+/−	++
4-Thiouracil	+++/−	++	++	+/−	+/−	+++/−	+++/−	+/−	+++/−
2-Methylthiouracil	+/−	+/−	+/−	+/−	+/−	+/−	+	+/−	+
2-Methylthiocytosine	+/−	+/−	+/−	+/−	+/−	+/−	+/−	+/−	+/−
3-Methyluracil	+/−	+	+/−	+/−	+/−	+/−	+/−	+/−	+/−

## Data Availability

The original contributions presented in this study are included in the article; further inquiries can be directed to the corresponding author.
